# 

**Published:** 1889-01

**Authors:** 


					﻿THIRTY-TWO EXTRA PACES IN THIS NUMBER.
Whole Number,
cccxxx.
January, 1889.
No. 6.
VOL. XXVIII
New Series.
Buffalo
Medical^Surgical
Journal.
Established. 1845 by AUSTIN FLINT, M. D.
Editors
THOS. LOTHROP, M. D.	W. W. POTTER, M. D.
Associate Editors
FLOYD S. CREGO, M. D. EDWARD B. ANGELL, M. D., Rochester, N.Y.
□
IB
I
®
J
(0
D
0.
0
z
H
I
r
<
BUFFALO:
1888.
Entered at the Post-Office at Buffalo, N. Y., as Second-class Mail Matter.
Times Printing House, Buffalo, N. Y.
				

